# Langerhans cell histiocytosis misdiagnosed as cow protein allergy: a case report

**DOI:** 10.1186/s12887-021-02921-8

**Published:** 2021-10-22

**Authors:** Xintong Lv, Libo Wang, Zhuang Pi, Chunyan Zhang

**Affiliations:** Department of Pediatric Gastroenterology, The First Hospital of Jilin University, No. 1 of Xinmin Street, Chaoyang District, Changchun, 130012 Jilin Province China

**Keywords:** Infant, Langerhans cell histiocytosis, Rash, Diarrhea, Cow milk protein allergy

## Abstract

**Background:**

Langerhans cell histiocytosis (LCH) is a heterogeneous disease with diverse clinical manifestations. Abdominal organ involvement is rare. Early diagnosis and active treatment are needed. The purpose of this article is to enable readers to have a better knowledge of LCH and prevent misdiagnosis.

**Case presentation:**

A 2-month-old boy had diarrhea, hematochezia, and a rash, and was diagnosed as having a cow milk protein allergy (CMPA). He was given an amino acid-based formula, but there was no sign of improvement in his condition.

The patient then had a skin biopsy and was diagnosed as having multisystem Langerhans cell histiocytosis (MS-LCH). The general condition of the child deteriorated after the first two doses of chemotherapy, and the child died.

**Conclusions:**

MS-LCH is a protracted and progressive condition with poor prognosis. Early diagnosis and treatment are essential for survival. If a child has chronic diarrhea and hematochezia in the presence of a characteristic rash, the pediatrician should consider the possibility of this disease to avoid misdiagnosis.

## Background

Langerhans cell histiocytosis (LCH) was considered to be a monoclonal dysplasia of Langerhans cells [1], but it is now believed to be the clonal proliferation of dendritic cells originating from bone marrow, with the infiltration of inflammatory cells. These dendritic cells are CD1a/CD207 positive. LCH is a broad spectral heterogeneous group of diseases with diverse clinical manifestations. Gastrointestinal tract (GIT) involvement in LCH has been very infrequently reported. GI manifestations of LCH are quite variable and unspecific. Bloody stools, intractable diarrhea, protein-losing enteropathy, constipation, abdominal pain, vomiting, malabsorption, and even intestinal perforation are some of the reported symptoms. Prognosis of children with gastrointestinal tract (GIT) involvement in LCH has been poor. Singhi et al. [2] reported 12 patients, including 2 children. One pediatric patient was lost to follow-up; however, the other patient was found to have concurrent systemic disease with skin and bone marrow involvement. The child died 1 year after initial diagnosis. Yadav SP et al. [3] reported 2 children and reviewed 37 other cases of LCH with GIT involvement, More than 50% patients died within 18 months from diagnosis. So early diagnosis and active treatment are needed. The purpose of this paper is to strengthen clinicians’ understanding of LCH.

## Case presentation

**Fig. 1:**
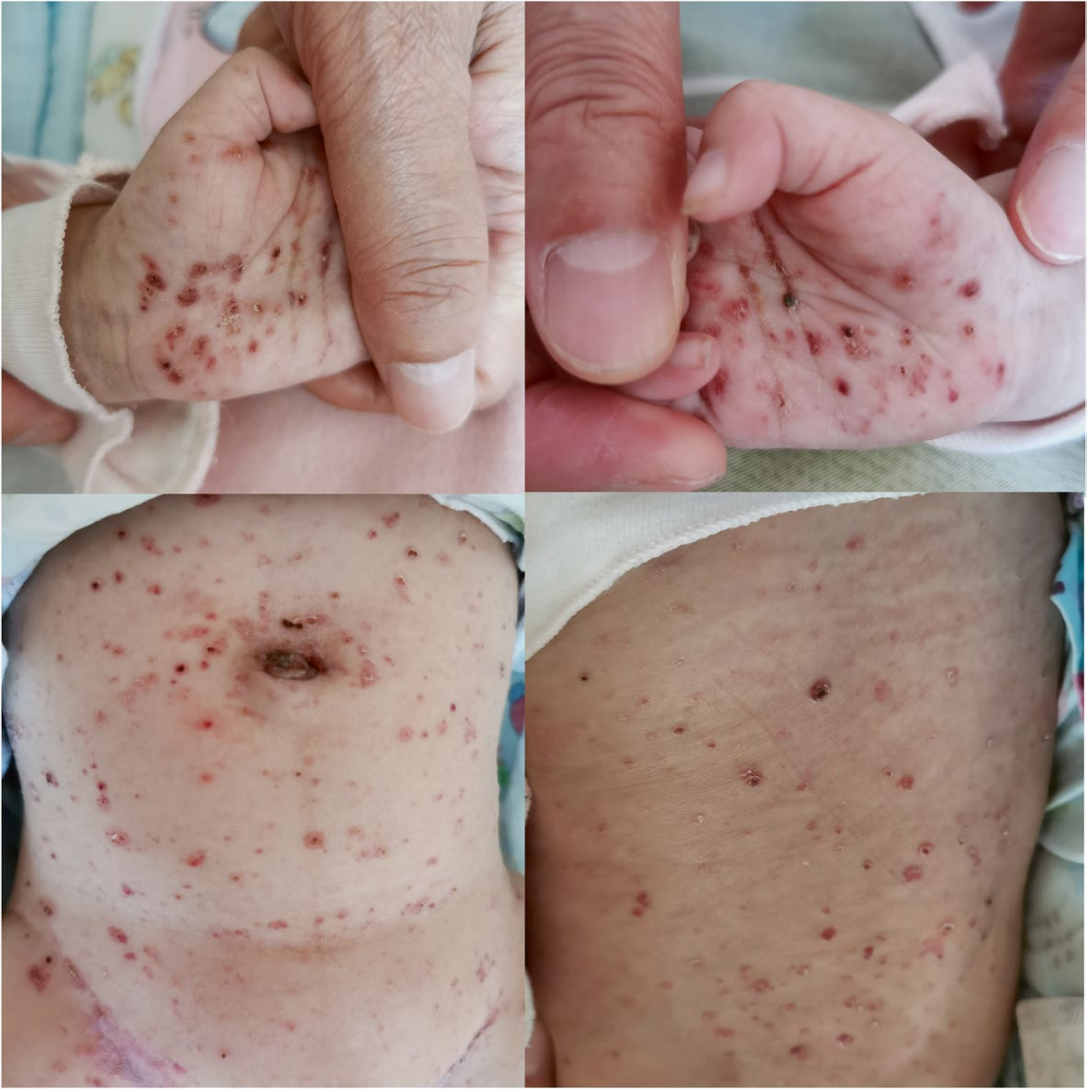
Physical examination. Papular rashes were seen in the face, trunk, limbs, scalp, perineum and palms. Some lesions were exudative with scab formation. Some coalesce and become confluent

**Fig. 2:**
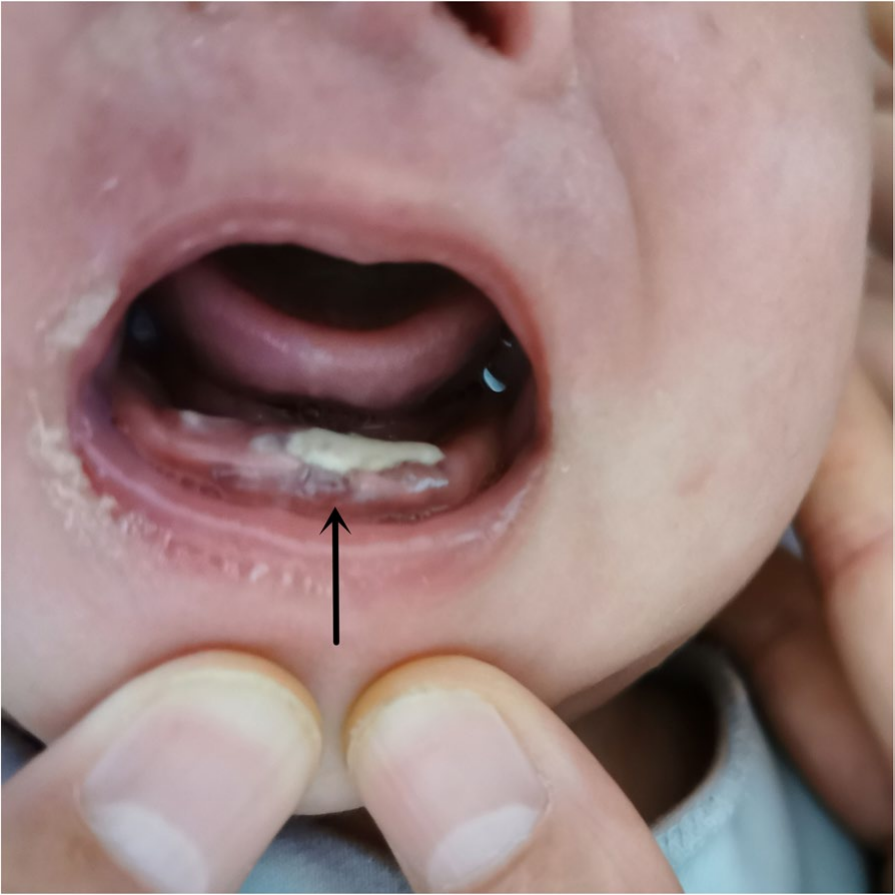
Mouth of the child. Ulceration with yellowish exudate were seen in the gums. Defect could be seen in the central mandibular gingiva (black arrow)

**Fig. 3:**
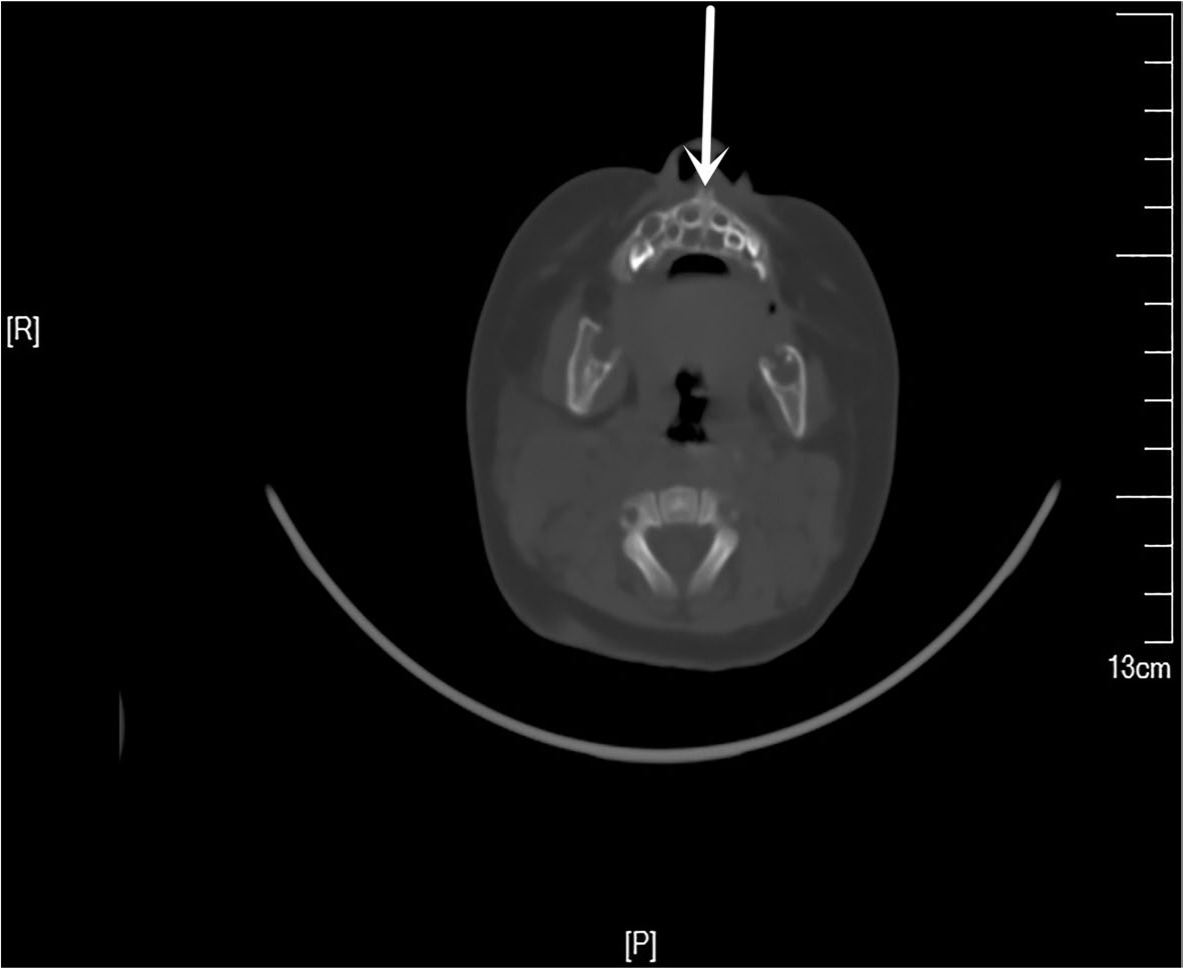
Brain CT scan of this child. The CT scan showed no obvious abnormal changes in the morphology and density of brain tissues, and the bilateral frontotemporal parietal subarachnoid space slightly widened. The position, size and density of cerebral ventricles and cisterns were not abnormal, and the midline structure was not displaced. The density of unerupted incisors and canine teeth were found decreased (white arrow)

**Fig. 4:**
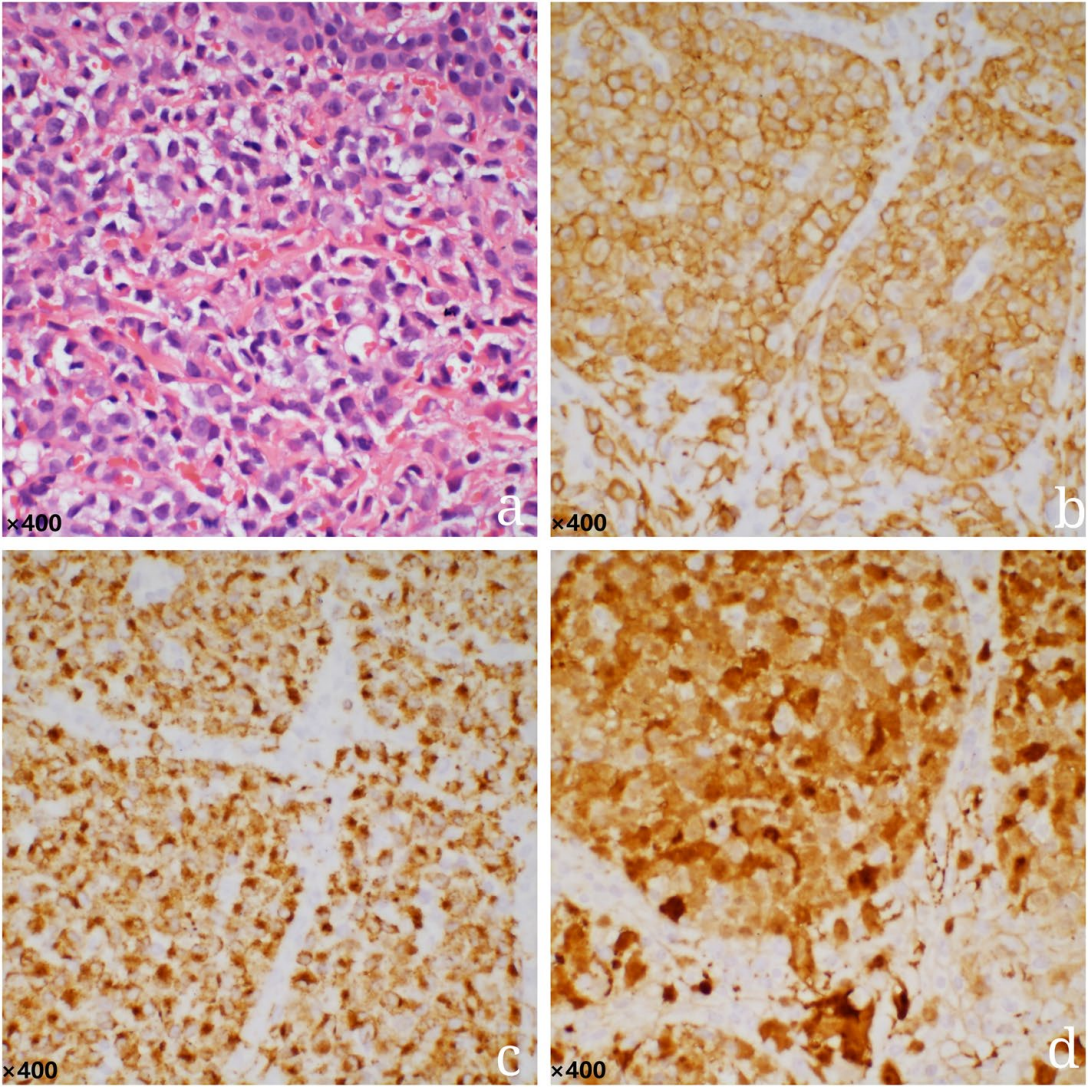
Dermal pathology showed: S-100 (+), CD1a (+), CD68 (+), Langerin (+), CD20 (−), Ki-67 (+ 40%), CD21 (−), CD23 (−), CD3 (focal +), Bcl-2 (focal +), CK-pan (−), EMA (−), HMB45 (−). **a** H-E staining of dermal pathology (× 40); **b** Immunohistochemical staining for CD1a (× 40); **c** Immunohistochemical staining for Langerin (× 40); **d** Immunohistochemical staining for S-100 (× 40). The resolution: a:1360*1024;b:1360*1024;c:1360*1024;d:1360*1024; These figures have not been processed to improve resolution. The scale bar of every microscopy image is 400

A 2-month-old boy was admitted to the hospital with a history of diarrhea, hematochezia, and rashes since his birth on June 4, 2019. His birth weight was 3.8 kg, and he weighed 6 kg when he was 2 months old (WHO Z score [4] 0–2). Initially, he was diagnosed as suffering from a cow milk protein allergy (CMPA) as he was being fed a cow milk protein-based formula. He was changed to an amino acid-based formula, but after 1 month there was no improvement. Physical examination revealed generalized rashes on the face, trunk, limbs, scalp, perineum, and palms (Fig. 1). The rashes were erythematous and papular, with scab formation. Several of them had become confluent and coalesced to form larger lesions. Yellow exudates and scab formation could be seen in the external auditory canals bilaterally, and superficial ulceration and yellowish white secretions were seen in the gums of the oral cavity (Fig. 2). The cervical, axillary, and groin lymph nodes could be palpated. A routine blood examination showed white blood cell 14.44 × 10^9^/L, red blood cell 3.92 × 10^12^/L, hemoglobin 112 g/L, and high-Sensitive C-reactive Protein 27.24 mg/L. A liver function test showed albumin 24.0 g/L, and the TBNK cell findings were total T cell (CD3+) 33.8%, CD4/CD8 4.45, and B lymphocyte (CD19+) 45.1%. A brain computed tomography scan showed decreased density of the unerupted incisors and canines (Fig. 3). The dermal histopathology results were obtained by the microscope OLYMPUS BX53 and cellSens Standard softwareas and as follows: S-100 (+), CD1a (+), CD68 (+), Langerin (+), CD20 (−), Ki-67 (+ 40%), CD21 (−), CD23 (−), CD3 (focal +), Bcl-2 (focal +), CK-pan (−), EMA (−), and HMB45 (−) (Fig. 4). The patient was diagnosed as having multisystem LCH (MS-LCH) (Grade IV). He was then transferred to the pediatric oncology department for chemotherapy. The general condition of the child deteriorated after the first 2 weeks of VP solution (prednisone and vinblastine), and he died.

## Discussion and conclusions

LCH can occur in any age group but it is mostly seen in children [5], and the incidence rate is 4.0–5.4/1,000,000. The pathogenesis of the disease remains unclear [6]. The lesion may involve multiple organs such as the bones (80%), skin (33%), pituitary (25%), liver, spleen, lungs, hematopoietic system (15%), lymph nodes (5–10%) and central nervous system (2–4%).

LCH rarely affects the digestive tract [7], but diarrhea is one of the first symptoms when it does. Up to now, there are only 5 literature reports about LCH with gastrointestinal involvement, and the age of 4 cases in these reports were younger than 2 years old. The clinical symptoms of LCH with gastrointestinal involvement include: nausea, vomiting, abdominal pain, diarrhea, hematochezia, constipation, intestinal obstruction, intussusception, and intestinal perforation. It can also occur in the distal ileum, making it difficult to differentiate from appendicitis.

Seborrhea or an eczema-like rash is seen in 86% of patients with LCH digestive tract involvement [8]. Therefore, a typical erythematous rash may be a clue to a diagnosis of LCH, and it is vital that it is differentiated from CMPA.

Bone, especially maxillofacial skeleton, is the organ most commonly involved in LCH. LCH occurs in the mandible more than the maxilla. The first symptoms of LCH with mandibular invasion are maxillofacial swelling, pain, restricted mouth opening, ulceration of gums or mucous membranes, teeth mobility or loss. In this case, superficial ulceration and yellowish white secretions were seen in the gums of the oral cavity, and surrounding mucosa was swollen. In order to assess the presence of skull and mandible invasion, the patient also underwent head CT examination. No skull involvement was observed, but the density of unerupted front teeth and canine teeth decreased, providing clues and basis for the diagnosis of LCH. In addition, in this patient, bilateral serous otitis was also a clue to a diagnosis of LCH.

It still needed to be differentiated from other diseases in this patient. 1) Late vitamin K deficiency. In this case, the child had the symptom of hematochezia, but his coagulation function was normal. And the child took formula milk, thus the diagnosis of vitamin K deficiency was not considered. 2) Intestinal infection. The child showed symptoms of diarrhea and hematochezia, but no white blood cells were found in routine stool test, and no pathogenic bacteria were cultured in stool microbiological test, so infectious diseases could be excluded. 3) Early-onset inflammatory bowel disease (IBD). The child had diarrhea and hematochezia symptoms, and the weight gain was not ideal. Therefore, the possibility of early-onsite IBD should be taken into consideration. However, since the parents of the child refused to undergo colonoscopy, early-onset IBD could not be completely excluded. Thus, LCH with GIT involvement was considered as final diagnosis based on the monistic explanation.

European guidelines say that a diagnosis of LCH should mainly depend on the histopathology of a biopsy. In this case, Langerhans cells were found in the skin biopsy, and the positive immunohistochemical findings of Langerin, CD1a, and S-100 proteins established the diagnosis of LCH [9]. Currently, LCH is divided into two groups, namely single system LCH (SS-LCH), with single organ or system involvement, and MS-LCH, with the involvement of two or more organs or systems. This patient was a 2-month-old infant with skin, lymph node, ear, and oral lesions, recurrent diarrhea, hematochezia, and decreased liver albumin. Therefore, this case was diagnosed as MS-LCH (Grade IV).

Some patients with SS-LCH may have spontaneous regression of the disease. However, in most patients with MS-LCH [10], especially infants, the clinical course is protracted and progressive, with poor prognosis [11]. Yadav SP et al. [3] pointed out that the mortality rate of patients with gastrointestinal involvement in LCH was 55.5%, which was higher compared with 7% of patients with single-system involvement and 40% of patients with multi-system involvement. In retrospect, the gastrointestinal tract should be considered as a high-risk organ that affects prognosis. Almost 40% of patients required second-line therapy and/or hepatocyte transplantation, suggesting a poor prognosis for gastrointestinal involvement in LCH [3]. For this reason, early diagnosis and treatment are essential.

In this particular case, the patient was misdiagnosed as having CMPA initially and was fed with amino acid milk formula for 1 month, which delayed his receiving the correct treatment and may have contributed to his eventual death. It is hoped that this case report will improve the clinical knowledge of LCH and help prevent misdiagnosis.

## Data Availability

The datasets used and/or analysed during the current study available from the corresponding author on reasonable request.

## Abbreviations

LCH: Langerhans cell histiocytosis
MS-LCH: Multisystem Langerhans cell histiocytosis
SS-LCH: Single system-Langerhans cell histiocytosis
CMPA: Cow Milk Protein Allergy
CT: Computed Tomography
